# Adipocyte alterations in endometriosis: reduced numbers of stem cells and microRNA induced alterations in adipocyte metabolic gene expression

**DOI:** 10.1186/s12958-019-0480-0

**Published:** 2019-04-15

**Authors:** Masoumeh Majidi Zolbin, Ramanaiah Mamillapalli, Sepide E. Nematian, Laura Goetz, Hugh S. Taylor

**Affiliations:** Department of Obstetrics, Gynecology and Reproductive Sciences, Yale School of Medicine, 310 Cedar Street, New Haven, CT 06520 USA

**Keywords:** Endometriosis, microRNAs, MiR, Fat tissue, Adipocytes, Let-7, miR-342, Stem cells

## Abstract

**Background:**

Endometriosis is an estrogen dependent, inflammatory disorder occurring in 5–10% of reproductive-aged women. Women with endometriosis have a lower body mass index (BMI) and decreased body fat compared to those without the disease, yet few studies have focused on the metabolic abnormalities in adipose tissue in women with endometriosis. Previously, we identified microRNAs that are differentially expressed in endometriosis and altered in the serum of women with the disease. Here we explore the effect of endometriosis on fat tissue and identified a role for endometriosis-related microRNAs in fat metabolism and a reduction in adipocyte stem cell number.

**Methods:**

Primary adipocyte cells cultured from 20 patients with and without endometriosis were transfected with mimics and inhibitors of microRNAs 342-3p or Let 7b-5p to model the status of these microRNAs in endometriosis. RNA was extracted for gene expression analysis by qRT-PCR. PCNA expression was used as a marker of adipocyte proliferation. Endometriosis was induced experimentally in 9-week old female C57BL/6 mice and after 10 months fat tissue was harvested from both the subcutaneous (inguinal) and visceral (mesenteric) tissue. Adipose-derived mesenchymal stem cells in fat tissue were characterized in both endometriosis and non-endometriosis mice by FACS analysis.

**Results:**

Gene expression analysis showed that endometriosis altered the expression of *Cebpa*, *Cebpb*, *Ppar-γ*, *leptin*, *adiponectin*, *IL-6*, and *HSL*, which are involved in driving brown adipocyte differentiation, appetite, insulin sensitivity and fat metabolism. Each gene was regulated by an alteration in microRNA expression known to occur in endometriosis. Analysis of the stem cell content of adipose tissue in a mouse model of endometriosis demonstrated a reduced number of adipocyte stem cells.

**Conclusions:**

We demonstrate that microRNAs Let-7b and miR-342-3p affected metabolic gene expression significantly in adipocytes of women with endometriosis. Similarly, there is a reduction in the adipose stem cell population in a mouse model of endometriosis. Taken together these data suggest that endometriosis alters BMI in part through an effect on adipocytes and fat metabolism.

## Summary sentence

Reduced adipocyte stem cell numbers and microRNA driven changes in adipocyte gene expression contribute to the metabolic alterations and low BMI in women with endometriosis.

## Background

Endometriosis is a gynecological disorder characterized by the deposition and proliferation of endometrial cells or tissue outside the uterine cavity [1, 2] that occurs in 10% of reproductive-aged women [3]. While the most widely recognized symptoms of endometriosis are pelvic pain [4] and infertility [5], which are reported in more than 50% of patients, endometriosis is also associated with symptoms unrelated to the reproductive tract [6]. Women with endometriosis have a lower body mass index (BMI) than those without the disease [7]. In a mouse model of endometriosis we have previously shown that endometriosis decreases weight gain and body fat levels [8]. Similarly, lipid dysfunction and fat loss have been reported in humans with endometriosis [9].

Adipose dysfunction is characterized by morphological alterations including subcutaneous fat atrophy, fibrosis and metabolic alteration [10]. To maintain normal adipocyte numbers and function, adipocytes contain a population of mesenchymal stromal progenitor/stem cells (MSCs). MSCs are progenitor cells that are part of the stroma surrounding the mature adipocyte and preserve tissue homeostasis by regulating the number of mature adipose cells [11]. Endometriosis is known to interfere with the normal stem cell trafficking to the uterus that is necessary for endometrial growth and repair [12]. Altered stem cell mobility and engraftment characterize this disease [13–16]. Endometriosis is very effective at recruiting stem cells and lesions act as a sponge to attract these cells [17]. The reasons for diffuse symptoms and the precise pathophysiology of endometriosis are still not well understood [18]; however, inappropriate stem cell trafficking and stem cell defects are part of the pathophysiology of this disease and could affect adipose stem cell trafficking as well [19].

MicroRNAs (miRNAs) are small noncoding RNAs that regulate gene expression and bind to the 3′-untranslated region (UTR) of target mRNA [20], thus inducing translational inhibition and/or mRNA degradation of protein-coding genes, and regulating various cell activities, such as differentiation, proliferation, apoptosis, and motility [21, 22]. MicroRNAs have been implicated in controlling MSC differentiation into adipocyte lineage and regulating cell proliferation [23]. Increased expression of a specific miRNA generally causes the repression of translation of the targeted mRNA, whereas decreases in miRNA expression exert the opposite effect [24]. MicroRNAs are known to be differentially expressed in the serum of women with endometriosis [25] and are considered to be potent regulators of gene expression [26]. In this way, they play a key role in the growth of endometriotic lesions. Previously, our laboratory has reported the upregulation of miR-342-3p [27] and downregulation of Let-7b [25, 28] in the serum of women with endometriosis. Although these studies demonstrated that serum miRNAs are potential biomarkers for endometriosis, it is unclear if they contribute to the systemic manifestations of the disease. Specifically, the mechanisms involved in inducing the clinically observed low BMI phenotype in endometriosis are unclear.

Of the microRNAs known to be altered in endometriosis, Let-7b and miR-342 are thought to play a role in metabolism. MicroRNAs such as Let-7b are known to regulate glucose metabolism [29], and metabolic functions of obesity-derived adipocytes [30]. MiR-342-3p is involved in adipose differentiation in conjunction with brown fat-specific transcription factors [31], lipogenesis [32], and browning of adipose tissue [33]. In this study, we aim to determine the effects of both Let-7b and miR-342-3p on adipose tissue from patients with endometriosis. Here we report the effect of these two miRNAs on gene expression in adipose cells and the effect of endometriosis on adipose stem cell populations in a mouse model of endometriosis.

## Materials and methods

### Study population

Fat specimens were collected from 20 women with endometriosis (EMS) and without endometriosis (non-EMS, *N* = 10 per group) who were treated at Yale-New Haven Hospital (New Haven, CT, USA). The study population was selected from patients who underwent laparoscopy for multiple benign indications including adenomyosis, myoma, pelvic pain, infertility, or endometriosis and the participants gave written informed consent. Institutional Review Board (IRB) approval was obtained from Yale School of Medicine (New Haven, CT, USA).

### Cell culture

Subcutaneous fat tissue in the abdominal region of the body was collected under sterile conditions from patients with and without endometriosis. The tissue was subsequently minced into fine pieces (1–2 mm), and then digested in buffer containing collagenase type 2 (0.83 mg/ml, #LS004174, Worthington Biochemical Corporation, NJ) in sterile Hank’s balanced salt solution (HBSS, Life Technologies, CA, #14185–052) with 3% bovine serum albumin (BSA), calcium chloride (1.23 mM), magnesium chloride (1.03 mM) and zinc chloride (0.83 mM) for 75 min at 37 °C. The cell pellet was collected by centrifugation at 300×*g* for 3 min, re-suspended in HBSS with 3% BSA, and filtered through a sterile 40 μm (BD Biosciences CA, #352340) nylon mesh filter. The cells were then re-suspended in the growth medium DMEM/F12 medium (Life Technologies) containing 10% fetal bovine serum (FBS) and antibiotics (2% anti-anti), and plated and cultured in a T75 tissue culture flask that was maintained at 37 °C and 5% CO2-atmosphere. After 48 h, the non-adherent cells were washed off with phosphate buffered saline (PBS) while the adherent adipocytes were grown to 75% confluence. After the third passage the cells were transfected with miRNAs.

### Transfection of miRNAs

Adipocytes obtained and cultured from controls without endometriosis were transfected with mimics, inhibitors and respective controls of miRNAs 342-3p and Let -7b-5p. Mimics are the mature microRNA sequences that result in functional microRNAs while inhibitors are the small nucleotide sequences that bind to functional microRNAs and inhibit their function. Both mimics (#SMM-003) and inhibitors (#SMI-003) were obtained from Bioneer Inc. CA, USA. Negative control (NC) is the scrambled sequence that does not show an effect on the target genes of a mature microRNA. Briefly, cells were trypsinized, centrifuged and plated in a 6-well plate at 2 × 10^5^ cells/well with 2 ml of growth medium without antibiotics. After 24 h the cells were transfected with the two miRNAs (50 nmol) mentioned above, using Lipofectamine™ 2000 and Opti-MEM® (Invitrogen, Carlsbad, CA) without serum or antibiotics according to manufacturer’s protocol. After 24 h the transfection media was replaced by growth medium with 10% FBS and antibiotics. Cells were cultured for an additional 72 h before RNA was extracted for gene expression analysis. Transfection efficiency was assessed by directly measuring the transfected microRNA in the cell lysate by qPCR. All transfections were carried out in duplicate wells under sterile conditions.

### Immunofluorescence studies

Immunofluorescence studies were carried out to determine PCNA expression as a marker of cell proliferation in both EMS and non-EMS groups. Cells were fixed at room temperature in 100% chilled methanol for 5 min, then washed three times with PBS and permeabilized with 0.25% Triton X-100 followed by blocking with 1% BSA in PBST (PBS + 0.1% Tween 20). Cells were incubated with anti-PCNA antibody (#ab18197, Abcam, 1:500 dilution) and anti-vimentin (#sc-373,717, Santacruz, 1:50 dilution) antibody in 5% BSA in PBST overnight at 4 °C. The next day, the cells were washed with PBS and incubated with secondary antibody in 1% BSA and counterstained with DAPI (46-diamidino-2-phenylindole; #H-1200; Vector Laboratories, Burlingame, CA). The PCNA antibody stains the cell nucleus while the vimentin antibody stains the cytoplasm.

Lipolysis was evaluated with hormone-sensitive lipase (HSL) staining (#4107 s, Cell Signaling Technology, Danvers, MA, 1:100 dilution). HSL protein levels determined in subcutaneous fat tissue sections were obtained from patients with and without endometriosis. Fat tissue from both groups was fixed in 4% paraformaldehyde, embedded in paraffin, and cut into 5-μm serial sections. Tissue sections were deparaffinized in xylene, rehydrated through a series of ethanol washes, then placed in boiling sodium nitrate (pH 6.0) followed by staining with anti-HSL antibody. Nuclear staining on sections was carried out using vectashield fluorescent mounting media with DAPI (Vector Laboratories). Negative controls were determined with the same protocol using its respective host protein IgG as an isotype control. All stained sections on slides were visualized under LSM 710 confocal microscopy (Carl Zeiss, New York, NY) using ZEN software (Carl Zeiss, New York, NY).

### Quantitative real time polymerase chain reaction (qRT-PCR)

Total RNA was extracted from post-transfected cells using TRIzol reagent (Invitrogen) and purified using RNeasy cleanup kit (Qiagen, Valencia, CA). For cDNA synthesis, purified RNA (1000 ng) was reverse-transcribed using iScript cDNA synthesis kit (Bio-Rad Laboratories, Hercules, CA). Real-time quantitative PCR (real-time qPCR) was performed using SYBR Green (Bio-Rad) and optimized in the MyiQ single-color real-time PCR detection system (Bio-Rad). Reactions were performed in a 10 μl volume containing 1 μl cDNA, 1 μl of primer mix, 5 μl master mix (SYBR® Green I; Applied Biosystems, Inc., Foster City, CA) and 3 μl water. PCR cycling conditions were as follows: polymerase activation and the initial DNA denaturation step required a temperature of 95 °C for 3 min followed by 40 cycles of 30 s denaturation at 95 °C and 20 s of annealing and extension at 57 °C. The specificity of the amplified transcript and absence of primer-dimers was confirmed by a melting curve analysis. Gene expression was normalized to beta actin (*ACTB)* as an internal control. Relative mRNA expression was calculated using the comparative cycle threshold (Ct) method (2^−ΔΔCT^). All experiments were carried out three times and each in triplicate. Primer sequences used for gene expression analysis are listed in Table 1 and were obtained from the W.M. Keck Oligonucleotide Synthesis Facility (Yale University, New Haven, CT).

**Table 1:** Sequences of primers used

HSL	TGCTAGGCACATAGCCTCCT	GCTGGGCTATGGGTGTCTTT	NM_005357.3
CEBPA	GCAAACTCACCGCTCCAATG	CTTCTCTCATGGGGGTCTGC	NM_004364.4
CEBPB	GCCGGTTTCGAAGTTGATGC	TTACACGTGGGTTGCGTCAG	NM_005194.3
Leptin	TGCGGATTCTTGTGGCTTTG	CTGACTGCGTGTGTGAAATGT	NM_000230.2
ADIPOQ	TTCCATACCAGAGGGGCTCA	CCCTTGAGTCGTGGTTTCCT	NM_004797.3
PPARG	CCGTGGCCGCAGAAATGAC	CACGGAGCTGATCCCAAAGT	NM_005037.5
ADIPONECTIN	TTCCATACCAGAGGGGCTCA	GAGTCGTGGTTTCCTGGTCA	NM_001177800
IL6	ACCCCCAGGAGAAGATTCCA	GTCTTCCCCCACACCAAGTT	NM_000600
β-actin	GAAGATCAAGATCATTGCTC	AACGCAACTAAGTCATAGT	NM_001101

### Endometriosis induction in mice and fat tissue collection

Endometriosis was induced experimentally in 9-week old female C57BL/6 mice (*n* = 5) (Charles River Laboratories, Wilmington, MA) with intact ovaries as previously described [34, 35]. Mice were housed in the Yale Animal Resources Center (YARC) at Yale School of Medicine and maintained (4–5/cage) in a room (21 ± 1C) with a 12-h light/dark cycle (7:00 a.m. to 7:00 p.m.) with ad libitum access to food (Purina Chow; Purina Mills, Richmond, IN, USA) and water. All animal experiments were conducted in accordance with an approved protocol from Institutional Animal Care and Use Committee (IACUC) of Yale University. IACUC guidelines were followed for animal care, surgery and for tissue collection. Briefly, donor uterine horns were split longitudinally to expose the lumen and endometriosis was induced by suturing two uterine fragments on the right and left peritoneal surface area using 5–0 polyglactin sutures (Ethicon, Somerville, New Jersey). Control female C57BL/6 mice (*n* = 5) underwent sham surgery placing sutures on the parietal pelvic peritoneum. Mice were sacrificed 10 months after endometriosis induction and fat tissue was harvested from both subcutaneous (inguinal) and visceral (mesenteric) areas. The tissue was immediately digested in digestion buffer at 37 °C for 1 h and single cell suspension was prepared for cell analysis by flow cytometry.

### Fluorescence-activated cell sorting (FACS) analysis

Adipose-derived mesenchymal stem cells in fat tissue were characterized in both endometriosis and non-endometriosis mice by FACS analysis. Single cell suspensions were prepared from fat tissue after digestion and stained with anti-CD140b APC/Cy 7 (#103027), anti-CD146 PE (#134703) and anti-LY-6A/E PE/CY5 (SCa-1, #108109) antibodies obtained from BioLegend, San Diego, CA. Antibodies were diluted according to the manufacturer’s recommendations (1:500) in 3% BSA/HBSS. The stroma vascular fraction (SVF) was re-suspended in the antibody staining solution, incubated on ice for 30 min, and washed and centrifuged at 300×g for 3 min. The SVF pellet was re-suspended, filtered through a 40 μm nylon filter and subjected to FACS analysis. Isotype-matched negative controls were used to define background staining. Cell sorting was performed using BD FACS Aria II software (BD Biosciences, Franklin Lakes, NJ).

### Statistical analysis

GraphPad Prism 7.0 (GraphPad Software for Science Inc., San Diego, CA) was used for all statistical analyses. Gene expression data are presented as mean ± SE (standard error). Statistical significance is determined based on two-tailed t-tests for gene expression and two-way ANOVA analysis for the stem cell comparisons. Student t-test was used for evaluating normally distributed variables. *P* < 0.05 was considered to be statistically significant.

## Results

### Altered adipocyte metabolic gene expression is regulated by microRNAs in endometriosis

Several microRNAs are expressed at altered abundance in women with endometriosis [28, 36]. To test the effect of miRNAs Let-7b and 342-3p on adipocytes, we transfected these miRNA mimics as well as their inhibitors in primary adipocyte cells cultured from fat tissue of women without endometriosis and examined genes known to be key mediators of adipocyte differentiation that are predicted targets of these microRNAs. Expression levels (mRNA) of CCAAT/enhancer binding protein alpha (*Cebpa*) and beta (*Cebpb*), peroxisome proliferator-activated receptor gamma (*Ppar-γ*), *leptin*, adiponectin (*Adipoq*), interleukin-6 (*IL-6*), and hormone-sensitive lipase (*HSL*) genes were analyzed by qRT-PCR.

**Fig. 1:**
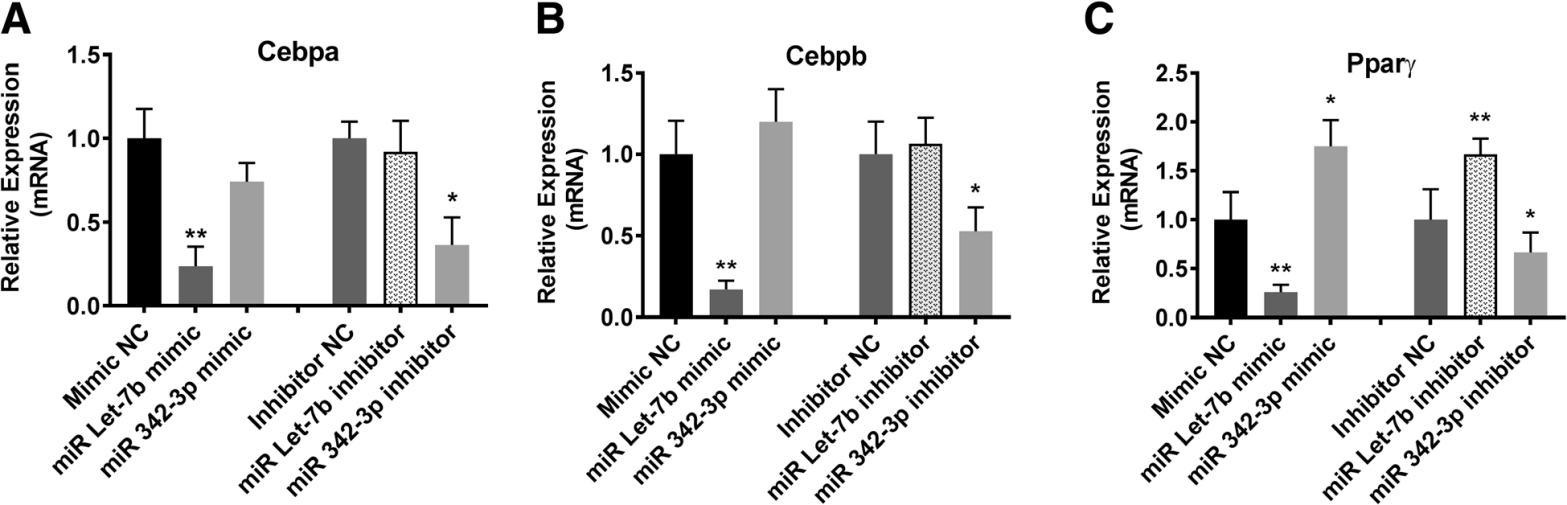
Altered gene expression in brown adipocyte differentiation. Let-7b mimic and miR-342-3p inhibitor significantly reduced mRNA levels of *Cebpa* (**a**), *Cebpb* (**b**), and *Ppar-γ* (**c**). *Ppar-γ* mRNA levels were increased by Let-7b inhibitor and miR 342-3p mimic, alterations similar to those seen in endometriosis**.** Each bar represents the mean ± SE for data from three individual experiments and each experiment was performed in triplicate. ** denotes statistical significance (*p* < 0.01) compared to the negative controls while * denotes *p* < 0.05

The Let-7b inhibitor treatment models the endometriosis state of reduced Let-7b expression, while the Let-7b mimic serves to increase Let-7b activity. Similarly, miRNA 342-3p mimic models endometriosis with increased levels, while the corresponding inhibitor serves to create the opposite effect as is seen in endometriosis. The Let-7b mimic significantly reduced the mRNA levels of *Cebpa*, (4.0 fold) *Cebpb* (5.1 fold), and *Ppar-γ* (3.2 fold) as shown in Fig. 1a, b and c respectively while miR-342-3p mimic increased the mRNA levels of *Ppar-γ* (1.75 fold) as shown in Fig. 1c compared to controls. Let-7b inhibitor increased *Ppar-γ* expression significantly (2.1 fold) as shown in Fig. 1c, while miR-342-3p inhibitor significantly reduced the expression of *Cebpa,* (2.4 fold, Fig. 1a), *Cebpb* (1.7 fold, Fig. 1b), and *Ppar-γ* (2.1 fold) as shown in Fig. 1a, b and c compared to controls.

**Fig. 2:**
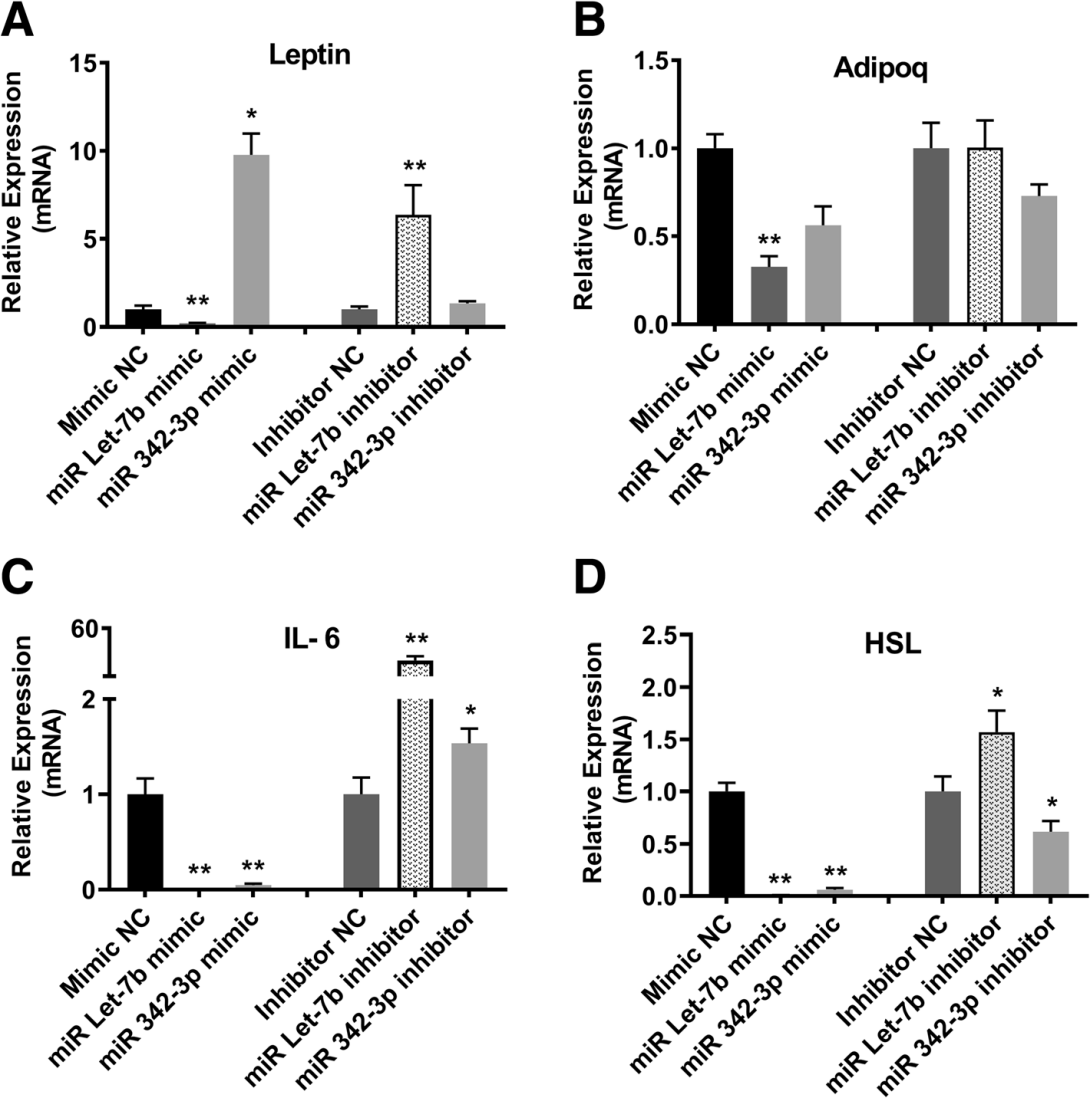
Altered expression of genes affecting appetite and insulin sensitivity. **a** *Leptin* mRNA levels were reduced by Let-7b mimic and increased by Let-7b inhibitor and miR 342-3p mimic. **b** *Adipoq* mRNA levels were reduced by Let-7b mimic*.* **c** *IL-6* mRNA levels were reduced by Let-7b and 342-3p mimics, and increased by Let-7b and 342-3p inhibitors. **d** *HSL* mRNA levels were reduced by Let-7b mimic and increased by Let-7b inhibitor, and decreased by both 342-3p mimic and inhibitor. Each bar represents the mean ± SE for data from three individual experiments and each experiment was performed in triplicate. * denotes statistical significance (*p* < 0.05) compared to the negative controls while ** denotes *p* < 0.01

As adipocytes are the main source of the *leptin* and *adipoq* secreted into blood in metabolic disorders, we tested the mRNA levels of *leptin* and *adipoq*. MiR Let-7b mimic reduced *leptin* mRNA levels (2.1 fold), while its inhibitor increased expression (6.37 fold, Fig. 2a). In contrast, *leptin* levels were increased after miRNA 342-3p mimic transfection (9.78 fold), and did not significantly change with its inhibitor (Fig. 2a). *Adipoq* expression was reduced by the Let-7b mimic (2.2 fold), and not significantly altered by any of the other transfections (Fig. 2b).

We additionally investigated the potential for these circulating miRNAs associated with endometriosis to alter adipocyte function by investigating their effect on the expression of *IL-6* and *HSL*, important mediators of fat metabolism. We found that *IL-6* expression was decreased in cells transfected with the Let-7b mimic (69 fold) and the 342-3p mimic (45 fold), while *IL-6* expression was increased by the Let-7b inhibitor (26.7 fold) and the 342-3p inhibitor (1.53 fold, Fig. 2c). *HSL* expression was also reduced by Let-7b mimic (92 fold), and increased by its inhibitor (1.57 fold), while both mimic and inhibitor of miRNA 342-3p decreased the levels of *HSL* by 6.5 fold and 1.62 fold, respectively (Fig. 2d). These findings indicate the potential for changes in Let-7b to drive increased fat metabolism in patients with endometriosis.

**Fig. 3:**
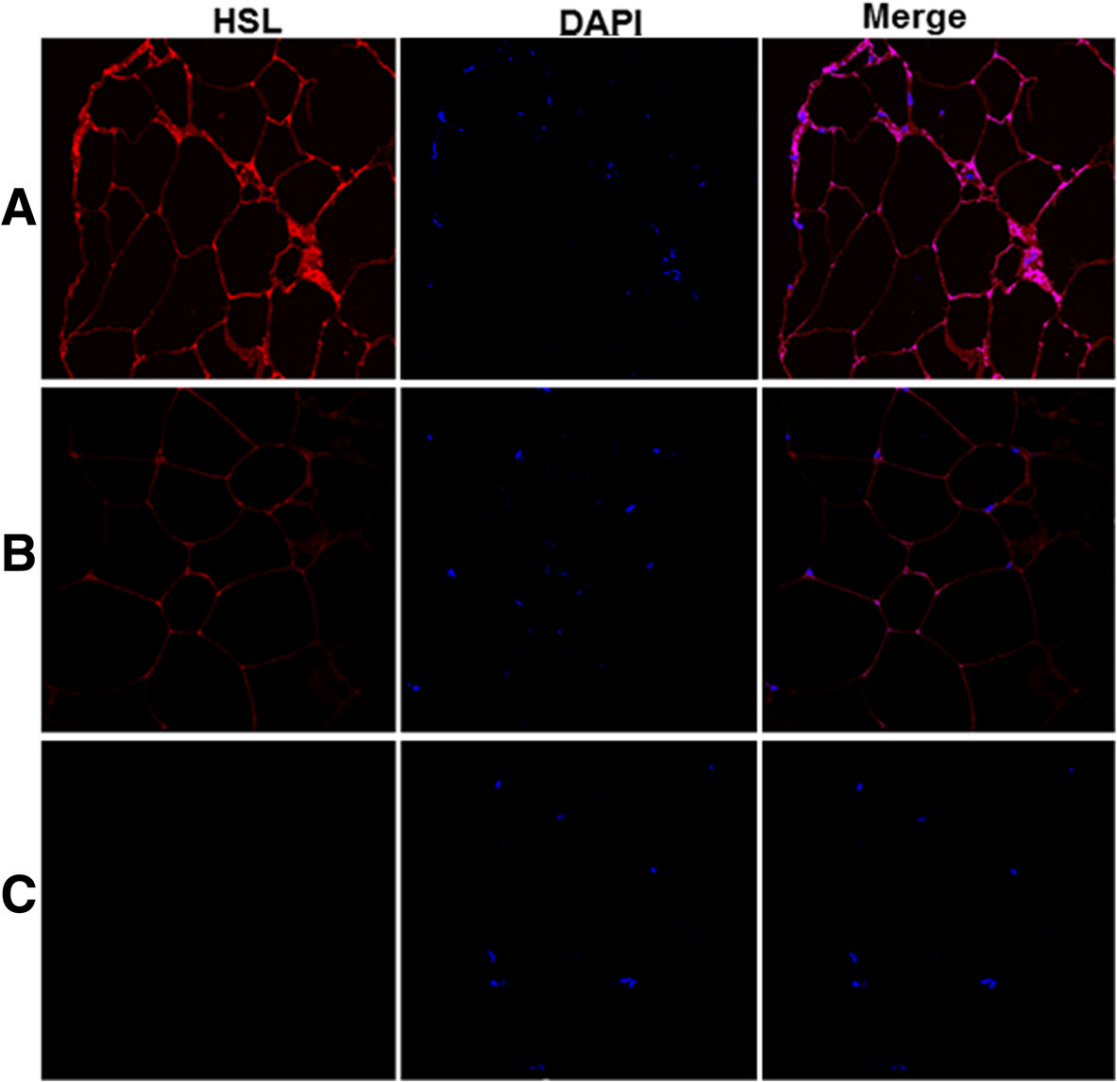
Analysis of hormone sensitive lipase (HSL) enzyme by immunofluorescence. HSL protein staining in tissue sections from subcutaneous fat: **a** EMS, **b** non-EMS and **c** Negative isotype control

To confirm that the *HSL* mRNA levels determined by qRT-PCR from cultured adipocytes mimicked the changes seen in vivo, we performed immunofluorescence (IF) staining studies for HSL protein levels in fat tissue collected from patients with endometriosis and without endometriosis. Subcutaneous fat tissue sections were subjected to IF using anti-HSL antibody. HSL staining is stronger in tissue from women with endometriosis (Fig. 3a) compared to those without endometriosis (Fig. 3b). There is no staining for HSL protein in isotype control (Fig. 3c).

**Fig. 4:**
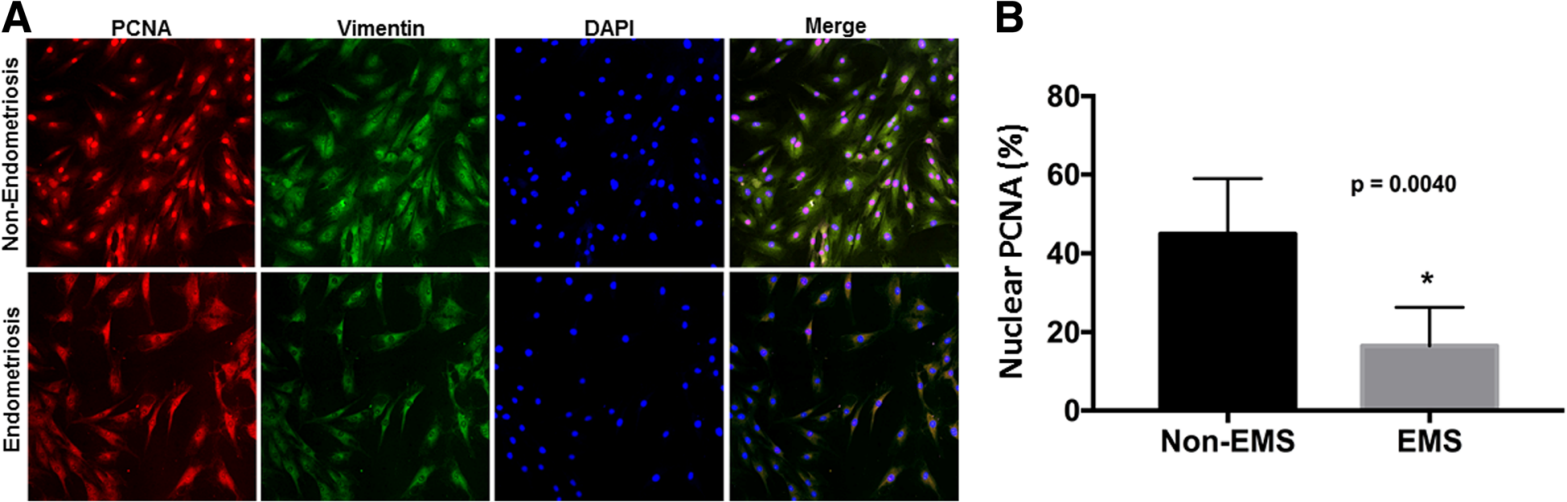
Analysis of PCNA expression by immunofluorescence. **a** Showing PCNA, and vimentin protein staining by their respective antibodies, with nuclear staining by DAPI in adipocytes from subcutaneous fat tissue of women with and without endometriosis. There is more vimentin protein staining in endometriosis patients than in non-endometriosis patients. **b** Reduction in nuclear PCNA protein expression in EMS compared to non-EMS. Each bar represents the mean ± SE. * denotes statistical significance (*p* = 0.0040) between EMS and non-EMS, (*N* = 10)

To gain further insight into the adipocyte cell proliferation and metabolism in subcutaneous fat from patients with and without endometriosis, we assessed the cultured adipocytes for expression of PCNA, a marker of cell proliferation, and for vimentin and DAPI simultaneously. Nuclear PCNA regulates proliferation, while the nuclear-to-cytoplasmic relocalization of PCNA is a key factor in shaping the energy metabolism [37–39]. Immunostaining results showed weaker nuclear PCNA and increased cytoplasmic staining in fat tissue of endometriosis patients compared to non-endometriosis patients (Fig. 4a). Also, we found that the number of cells stained for nuclear PCNA was significantly reduced (*p* = 0.004) in endometriosis patients compared to non-endometriosis as shown in Fig. 4b. While the majority of staining was nuclear in adipocytes cultured from women without endometriosis, the majority of PCNA was cytoplasmic in cells from endometriosis patients.

### Adipocyte stem cells (mASCs) are reduced in a mouse model of endometriosis

**Fig. 5:**
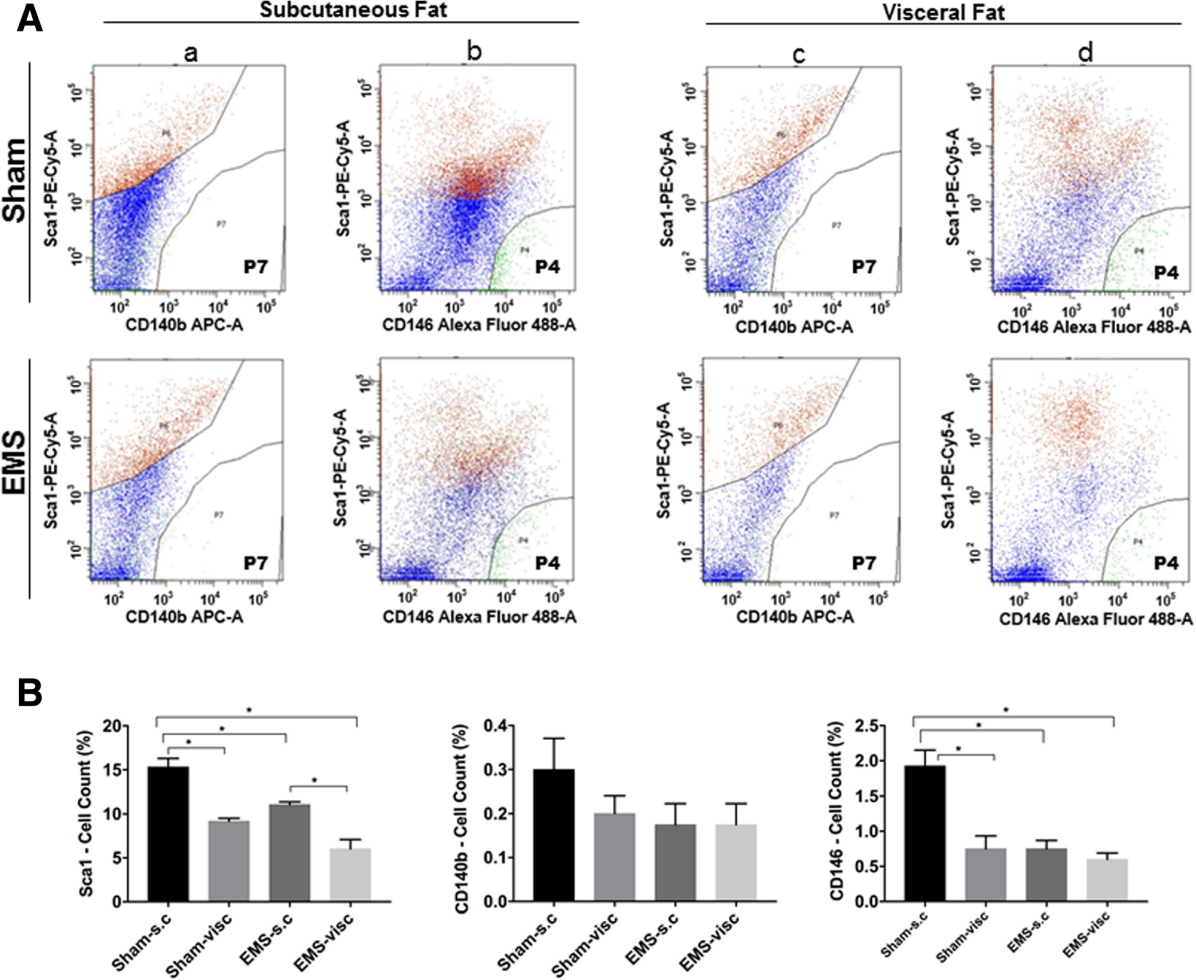
Analysis of mesenchymal stem cell markers by flow cytometry. **(A)** Flow cytometry histograms. **(a)** and **(b)**: subcutaneous fat; **(c)** and **(d)**: visceral fat from sham and EMS mice. Mesenchymal stem cell markers: Sca-1^+^, CD140b^*+*^, CD146^+^ . **(B)** Quantification of mesenchymal stem cell markers by flow cytometry. The number of Sca-1^+^ and CD146^+^ expressing stem cells are significantly lower in sham-visc, EMS-s.c and EMS-visc groups compared to sham-visc. EMS-visc has low Sca-1^*+*^ population compared to EMS-s.c group. Each bar represents the mean ± SE for data from three individual experiments and each experiment was performed in duplicate. * denotes statistical significance (*p* < 0.05) between sham-s.c vs other groups, EMS-s.c vs EMS-visc (Sca-1^+^)

To determine whether endometriosis affects the stem cell properties of fat tissue, we performed fluorescence activated cell sorting (FACS) to quantify the number of adipocyte stem cells. A mouse model was used to obtain sufficient cells from multiple fat compartments that are not easily accessed in humans. Subcutaneous and visceral fat tissue from both groups (endometriosis and non-endometriosis) was separately harvested and digested. Isolated cells were stained for adipocyte stem cell markers including Sca1, CD146*,* and CD140b. FACS analysis showed that more stem cells were present in subcutaneous and visceral fat in the non-endometriosis group than in the endometriosis group (Fig. 5A). The percentage of stem cells for each marker was calculated. Stem cells with Sca1^+^ and CD146^+^ markers were significantly higher (Sca1^+^*, p* = 0.0093; CD146^+^, *p* = 0.0013; Fig. 5B) in the subcutaneous fat of the non-endometriosis group than in the corresponding endometriosis groups. The same pattern was observed for visceral fat when using the Sca1^+^ marker. In general, the number of stem cells present in subcutaneous fat is higher than in visceral fat, and the number of stem cells with the Sca1^+^ marker is significantly reduced (*p* < 0.05) in all other groups compared to the sham subcutaneous group. Also, a significant difference was observed between the subcutaneous and visceral fat of the endometriosis group for the Sca1^+^ marker. No significant change was observed in stem cells with CD140b^+^, a marker for platelet-derived growth factor receptor (PDGFR), between endometriosis and sham groups, and between subcutaneous and visceral fat. There was also a change in the endothelial marker CD146 in either subcutaneous fat in the endometriosis group, indicating that the vascularity of the fat tissue may have also been impacted. Among all three stem cell markers analyzed, we observed the most significant change in the Sca1 marker between the sham and endometriosis groups in both subcutaneous and visceral fat of endometriosis.

## Discussion

Endometriosis is an estrogen dependent disease in reproductive-aged women that causes pelvic pain and infertility. Recent evidence shows that it is a systemic disease [40]. The systemic manifestations of endometriosis are, at least in part, mediated by abnormal levels of circulating miRNAs in women with the disease [27]. A recent review has thoroughly discussed the role of various microRNAs in the pathology of endometriosis [41]. Though several microRNAs are involved in endometriosis, including miR-125b-5p, miR-342-3p, miR-Let-7b, miR-3613-5p and miR-150-5p, this study focused on those thought to alter metabolism. However, we can not rule out the possible role of other microRNAs which were not tested in this study.

Here we demonstrate that miRNAs Let-7b and 342-3p, which we have previously shown to be differentially expressed in the serum of patients with endometriosis, influenced adipocyte gene expression. Inhibition of Let-7b and upregulation of 342-3p models the down-regulation and upregulation of these two miRNAs, respectively, as is seen in endometriosis patients. These miRNAs induced consistent changes in adipocyte metabolic gene expression that would be expected to dramatically change metabolism in these cells. The alterations would be expected to promote the clinically observed low BMI phenotype seen in endometriosis patients.

MicroRNA alterations associated with endometriosis affect genes such as *Cebpa, Cebpb,* and *Ppar-γ,* which are involved in brown adipocyte differentiation, *leptin* and *adipoq,* which are involved in glucose metabolism, and *IL-6* and *HS*L, which are involved in fat metabolism. MicroRNA 342-3p has been previously shown to have a role in brown adipocyte differentiation. Here we show that it regulates *Ppar-γ*, a transcription factor that has previously been implicated in the conversion of white adipose tissue to brown adipose tissue [42, 43]. *PPAR-γ* has also been shown to regulate fatty acid and glucose metabolism. Its expression is decreased in obese individuals [44], but increased in mice with endometriosis compared to mice without endometriosis [8]. *HSL* has been shown to drive lipolysis [45]. Our data suggest that decreased miR-Let-7b significantly decreased *HSL* expression in human fat. Taken together, our data indicate that the circulating miRNA profile associated with endometriosis may promote browning of adipose tissue.

These findings are consistent with the results reported by Goetz et al. showing an increase in *leptin* and *ppar-γ* expression in a murine model of endometriosis [8]. Leptin is a protein secreted by adipocytes and has been widely shown to improve insulin sensitivity and suppress appetite [8, 46–48]. Adipoq is a fat-derived hormone that has been demonstrated to improve insulin sensitivity, and is involved in brown adipose differentiation pathways as well [49–52]. Circulating miRNAs associated with endometriosis may drive appetite reductions and improvements in insulin sensitivity. Similarly, miR-342-3p up-regulation has been demonstrated with metformin treatment [53]. IL-6 is a cytokine that is secreted by adipose tissue. Since it has been shown to specifically stimulate fat metabolism, it is considered to be a lipolytic factor [54, 55].

We identified a lower proliferative ability of fat cells from endometriosis patients. Adipose tissue harvested from endometriosis patients showed alterations in cell proliferation corresponding to decreased nuclear PCNA expression. Interestingly, nuclear-to-cytoplasmic relocalization of PCNA has been demonstrated in non-proliferating cells; cytosolic PCNA is a key factor in shaping the energy metabolism, enhancing glycolysis [37, 38, 56]. Direct interaction between PCNA and NAMPT in the cytoplasm modulates the intracellular NAD+ level and glycolysis [39]. We identified a shift to cytosolic PCNA in endometriosis that likely also increases glycolysis, thus altering availability of lipid precursors. Longer doubling times and decreased nuclear expression of proliferation marker PCNA have also been associated with miR-342-3p in other conditions [57, 58]. Similarly miR-Let-7b acts as a key regulator of cell proliferation of stem cells [59].

Finally, we identified a reduction in adipocyte stem cells in the subcutaneous fat in a mouse model of endometriosis. We identify a dysfunctional phenotype of endometriosis adipose stem cells (ASCs) which can alter their differentiation and proliferation in the process of tissue regeneration. A reduction in Sca1 positive stem cells may interfere with fat cell expansion in endometriosis.

In conclusion, we have demonstrated for the first time significant alterations in the adipocytes of women with endometriosis. Fat cells have altered lipid and glucose metabolism as that predict a propensity toward lower fat mass and explain the lower BMI seen in women with endometriosis. The altered circulating microRNAs found in women with the disease affect adipocyte gene expression, and may be a mechanism by which endometriosis lesions communicate with fat leading to altered fat metabolism in these women. Further the decreased adipocyte stem cell numbers also suggest a second mechanism driving decreased adiposity. Endometriosis has widespread manifestations affecting multiple tissues. These include a lower BMI and less body fat; understanding the molecular and cellular etiology of this disorder may provide new targets for treatment and the ability to limit the systemic effects of the disease.

## Abbreviations

*ACT*: Beta actin
Adipoq: Adiponectin
BMI: Body mass index
BSA: Bovine serum albumin
CD146: Cluster of differentiation 146
Cebpa & Cebpb: CCAAT/enhancer binding protein alpha & beta
EMS: Endometriosis
non-EMS: Non-endometriosis
FACS: Fluorescence-activated cell sorting
HSL: Hormone-sensitive lipase
IL-6: Interleukin-6
miRNAs: MicroRNAs
MSCs: Mesenchymal stromal progenitor/stem cells
PBS: Phosphate buffered saline
PCNA: Proliferating cell nuclear antigen
PCR: Polymerase chain reaction
Ppar-γ: peroxisome proliferator-activated receptor gamma
qRT-PCR: Quantitative real-time polymerase chain reaction
RNA: Ribonucleic acid
UTR: 3′-untranslated region
